# The role of vertically integrated learning in a rural longitudinal integrated clerkship

**DOI:** 10.1186/s12909-019-1767-8

**Published:** 2019-09-03

**Authors:** Jessica Beattie, Marley Binder, Vivienne Ramsbottom, Lara Fuller

**Affiliations:** School of Medicine, Rural Community Clinical School, Deakin University, Geelong, Australia

**Keywords:** Vertically integrated learning, Longitudinal integrated clerkships, Rural medical education, General practice, Community based education, Qualitative research, Rural generalist pathway, Communities of practice, Near-peer learning

## Abstract

**Background:**

Deakin’s Rural Community Clinical School (RCCS) is a Longitudinal Integrated Clerkship (LIC) program in Western Victoria. Students undertake a year-long placement in a rural General Practice, many of which also host General Practice Registrars. There is a lack of evidence addressing the role and impact of Vertically Integrated Learning (VI) in practices hosting both LIC medical students and General Practice Registrars.

The objective of the study was to establish how VI is perceived in the LIC context and the impact that it has on both learners and practices, in order to consider how to potentiate the role it can play in facilitating learning.

**Methods:**

Semi-structured, in-depth, qualitative interviews were undertaken, with 15 participants located in RCCS General Practices. Emergent themes were identified by thematic analysis.

**Results:**

Five main interconnected themes were identified; (i) understanding and structure, (ii) planning and evaluation, (iii) benefits, (iv) facilitators, and (v) barriers.

**Conclusion:**

VI in a rural LIC is not clearly understood, even by participants. VI structure and methodology varied considerably between practices. Benefits included satisfying and efficient sharing of knowledge between learners at different levels. VI was facilitated by the supportive and collegiate environment identified as being present in a rural LIC context. Resources for VI are needed to guide content and expectations across the continuum of medical training and evaluate its role. The financial impact of VI in a rural LIC warrants further exploration.

## Background

Over time, a range of changes in the medical education and training environment have impacted on the number of learners undertaking concurrent medical training in Australian rural General Practices [1–3]. Firstly, there has been a paradigm shift in medical education, which increasingly recognises the importance of medical trainees at a range of levels gaining adequate experience in primary and community care settings [1–3]. Concurrently, in Australia, the important role of rural training time has been recognised as part of a strategy to address the workforce maldistribution that disadvantages rural areas [4, 5], with evidence to support extended rural training as an effective rural workforce recruitment strategy [6, 7].

Longitudinal Integrated Clerkships (LICs), in which medical students spend an extended period of training time attached to a General Practice (Family Medicine) and primary care context, have been a successful educational model implemented in Australia, encompassing both extended rural and primary care exposure [8, 9]. In LIC programs students complete longitudinal clinical attachments under the supervision of a nominated clinical preceptor and undertake an integrated learning model where they participate in comprehensive patient care over time [8, 10, 11].

In Australia, these changes within training programs have resulted in a steady increase in the numbers of trainees who are co-located in rural General Practices [12]. It is not uncommon for practices to simultaneously accommodate medical students, prevocational trainees on rotation and vocational trainees undertaking the General Practice training program. As a result, there has been a need for the development and adoption of efficient approaches in terms of both time and resources to simultaneously educate multi-level learners [12–15]. Vertically integrated learning (VI) is one such approach [3, 16].

VI has been defined as ‘(the) coordinated, purposeful, planned system of linkages and activities in the delivery of education and training throughout the continuum of the learner’s stages of medical education’ [16]. VI recognises that opportunities exist to develop and foster educational links between the various stages of training [14, 15]. Importantly, it highlights the potential for shared learning to occur between co-located trainees, and recognises the multidirectional flow of teaching and learning that occurs within a practice environment, with all parties recognised both as a learner and a teacher [17].

Theoretically, a LIC program is an environment in which a VI learning model should flourish, as the model promotes the embedding of medical students’ clinical learning within a community of practice, with multiple levels of learners in which they are recognised as a legitimate member of the clinical team and increasingly become more ‘active’ participants in patient care over time [18–21]. Longitudinal learning relationships are fundamental to LIC and VI models [18, 22]. At their core both are learner-centred models, with an emphasis on teaching and learning activities that provide flexibility and are responsive to the needs of the learners [3, 23].

Deakin University’s Rural Community Clinical School (RCCS) offers a 12 month comprehensive rural LIC program to 20 students in their penultimate year of Deakin’s’ 4 year graduate entry medical degree [8]. Following 2 years of pre-clinical training at a large regional campus, RCCS students are placed in groups of 2–4 students across nine rural sites (geographical classification ASGC RA 2–4) [24] in South West Victoria, where they are attached to a rural General Practice and its associated health service. In this primary care environment, students participate in all the core year three disciplines of medicine, surgery, musculoskeletal medicine, mental health, women’s and children’s health in a simultaneous, integrated way [8, 11]. The General Practices in which RCCS students are placed also host a number of vocational GP trainees for periods of 6–12 months.

Despite the natural alignment of LIC and VI philosophies, there has been little investigation into how VI is understood, recognised or conducted within LIC programs. This study aimed to explore whether and how VI is occurring in the General Practices that host Deakin University’s’ RCCS LIC students, and how it is perceived by those involved in it.

## Method

### Participants and recruitment

Participants in this study were learners from General Practices hosting RCCS LIC students.

Learners were defined as GP supervisors (preceptors), GP registrars (trainees), practice managers and medical students [3]. All learners situated within the RCCS General Practices were invited to participate in this study. None of the RCCS General Practices during the research period were involved in teaching prevocational trainee doctors.

Forty-eight potential participants (supervisors, practice managers and students) from 13 practices were contacted via email and invited to participate. The practice managers of participating General Practices promoted the project to their GP registrars. Participants were provided with a Plain Language Statement outlining the purpose and requirements of the study and asked to contact the research team if they were willing to participate. Fifteen participants, representing eight of the RCCS General Practices, contacted the research team to arrange interviews (Table 1).

**Table 1:** Types of learners

Learner	Number	General practices represented (A, B, C, D, E, F, G, H)
General Practice supervisors^a^	5	A, B, C, D, H
Practice Managers	2	A, B
Medical Students	5	A, C, E, F, G
General Practice registrars^b^	3	A, D
Total	15	8

^a^Equivalent to Family Medicine preceptors

^b^Equivalent to Family Medicine trainees

### Data collection

Participants partook in a qualitative in-depth semi–structured interview either face to face or via telephone. The qualitative methodology employed was a phenomenological approach, as this provides both an in-depth understanding of perception and meaning [25, 26]. The research script was developed using a reflexive approach based on the researchers’ prior knowledge and experience of the RCCS program [27]. The interview script was piloted to assess the appropriateness of the language used and determine whether topics elicited responses relevant to the research questions, with amendments made as required (Table 2) [28]. Interviews were conducted between May and August 2017. All interviews were conducted by the primary investigator (JB), audio recorded and transcribed verbatim [30, 31]. Participants provided verbal consent as approved by ethics, acknowledging they had read and understood the Plain Language Statement. After interviews were transcribed they were imported to qualitative software program NVivo 11 to supplement analysis [29–31].

**Table 2:** Interview script; topics and prompts

Topics	Prompts
Definition/understanding of VI	Levels of learners in the clinic
Types of VI in LIC	Frequency?
	Format?
	Level of learners?
	Favoured teaching or learning format?
Benefits of VI in an LIC	Why?
	How?
	What do you personally gain from this style of learning?
Disadvantages/barriers to VI	Why?
	Do you believe VI meets the needs of all learners?
Resources/or assistance for VI	Why?
	How?
	How do you evaluate VI?
	Have you implemented quality improvement strategies after evaluation?

### Qualitative analysis

Two researchers (JB and MB) independently thematically coded interviews. Interviews were coded line by line, by listening to interviews and reading transcripts. Once coded, researchers (JB and MB) discussed initial major and sub themes to develop a tentative thematic framework. The entire research team (JB, MB, LF and VR) then discussed the themes, reaching a consensus and adopting the thematic framework that was employed over the entire dataset [29, 30]. From the emergent themes key participant’s quotes were then extracted to illustrate the results found.

### Ethical approval

The project (**HEAG-H 196–2016**) was approved by the Deakin Human Ethics Committee in December 2016.

## Results

The study identified five interconnected major themes, with associated sub-themes; (i) understanding and structure, (ii) evaluation and planning, (iii) benefits, (iv) facilitators and (v) barriers.

### Understanding and structure

#### Perception

Supervisors and practice managers had a solid understanding of VI, believing that everyone is a learner, both learning from and teaching one another. Registrars and students were less familiar with VI terminology, with many stating they were unsure of what the definition was but associating it with the co-location of learners at different stages of training. Their comments also reflected the idea of a vertically integrated curriculum for learning.

*“Vertical integrated learning in my view is where you have ….professional people with different stages of their career sharing information with each other upwards and downwards, so basically you have learning which is bi-directional, multidirectional and taking into consideration different experiences and knowledge of the people involved so …it’s not a one way flow of information but both upwards or downwards of information.” (GP Supervisor 2)*

*“ I guess what I understand it to be, is kind of the spiral of learning that you know you learn a certain amount about a topic but then you kind of keep visiting it” (Medical Student 1)*

#### Structure

Participants primarily identified group learning sessions involving multiple levels of learners as examples of VI. The majority of General Practices held formal VI group learning sessions weekly for 1–2 h. Learners involved included supervisors, registrars, students and infrequently practices nurses. The format of these sessions ranged from presenting to the group, discussion of cases, or role play scenarios. Participants also identified parallel consulting [32] and learning that was present outside of the practice, for example ward rounds and opportunistic teaching from clinicians as other instances of VI.

*“So the first and probably the one I think works the best is where we have a group session where we spend 90 minutes together once a week and every participant is delegated a task and the task is about a theme or sometimes a clinical skills so it might be related to patients that somebody has seen.” (GP Supervisor 1)*

*“It’s (VI) primarily through parallel consulting which I do two half days of each week.” (Medical Student 3)*

#### Heterogeneity

Learning opportunities varied between RCCS general practices. Students reflected on their experiences, frequently making comparisons with their peers. Some perceived they had fewer VI learning opportunities than other LIC students due to limited group learning sessions. Other students reflected on the absence of learning opportunities associated with hospital clinical schools such as didactic formats.

*“I suppose you miss on the bigger hospital …I’ve heard about the other clinical schools, (and) we have a lot less formal tutorials. I personally like (this) but for the learners that just absorb like sponges they might prefer…the actual guidance, someone telling them all the information rather than having to find out, having to do a lot more on your own.” (Medical Student 3)*

*“I know at some of the other Practices throughout the RCCS sites, the students have really pushed for more formal structured teaching whereas we (students) are a lot more self-directed and not only that but (its) whether or not GP Supervisors want to set aside some time for formal teaching.” (Medical Student 2)*

### Evaluation and planning

#### Evaluation

There was an absence of evaluation processes regarding VI within the general practices, with practice managers and supervisors acknowledging this was an area where they would like further guidance. Reliance on formative and summative assessment results and informal anecdotal feedback were described as common evaluation measures, although these were not specific to evaluation of VI sessions.

*“I would love to be able to evaluate it and include the learners in some form of research as well to see what their subjective experience is of learning in a vertical integrated setting.” (GP Supervisor 1)*

#### Resources

There were no formal resources specific to planning format or content of VI sessions utilised, but participants indicated they would use these if they were available. Supervisors had often accumulated or developed their own teaching resources over many years. When tasks were allocated for group learning sessions, learners described using their own initiative regarding presentation content, which was not necessarily aligned with meeting each learner’s curriculum objectives. It was suggested that a resource outlining the learning objectives for different levels of learners, associated with different topics would be beneficial to teaching and planning.

*“I’m not sure if there is any solid (VI) resources, no.” (GP Supervisor 5)*

*“It would be useful if there was little packages involved for certain learning topics which actually, not to say with all the information in it but just to predesigned learning objectives for topic X is there already outlined as an available resource and just something we can use as a framework to ensure we are covering the requirements for all the different learners in the group. Just making sure we get everything out of it, so just a framework for each topic in terms of learning objectives.” (GP Supervisor 3)*

#### Time

Sufficient time to adequately plan sessions was identified as a barrier. Due to competing needs within the practice, sometimes sessions were conducted with no predetermined topic or learning objectives. Having to acquit the reporting requirements for multiple levels of learners, who belong to different training bodies, was also acknowledged as being very onerous.

*“I guess the major disadvantage that I encounter is a bit of an artificial one in as much as for the purposes of the training organisation, doing individual reporting, tracking the learning activities.” (GP Supervisor 4)*

*“It is time consuming, it does take time so time has to be taken away from consulting.” (GP Supervisor 2)*

### Benefits

#### Knowledge transfer

Overall the majority of participants described numerous benefits associated with VI. A frequent theme was the opportunity to learn from each other, with supervisors feeling they learnt as much from students and registrars as they taught them, and were kept abreast of current information. Participants felt VI highlighted each level of learner’s unique strengths: students’ knowledge of pathophysiology, registrars’ knowledge of current clinical guidelines/research and supervisor’s experience and clinical acumen.

*“I learn quite a lot from them (medical students and GP registrars) because their knowledge is far more up to date than mine would be. If I didn’t run sessions I would probably have to spend a lot more time having to keep up to date myself which I don’t really have time for.” (GP Supervisor 4)*

*“I guess some of the benefits are: you’re getting the information from different levels …you are getting some from the Registrars and then again from the GP but I guess at that next tier of knowledge…. we’ve learnt something new in lecturers they might not have seen that or heard about it, so it’s useful to them as well.” (Medical Student 4)*

#### Satisfaction

Supervisors and practice managers spoke of the satisfaction they gained from teaching students and registrars, taking particular pride in the fact they were helping to train the future medical workforce.

*“There is also the satisfaction of mentoring someone and the satisfaction knowing that you’re helping shape future doctors.” (GP Supervisor 2)*

*“It really makes me feel good when I hear the interaction and I hear the laughter and I hear the learning and the conversations and even if they disagree there is still that comradery.” (Practice Manager 1)*

#### Efficiency

VI was frequently commended for being an efficient model of teaching. Due to time and financial constraints the ability to operate as a teaching practice was only possible using VI tutorial formats

*“I actually think it’s (VI) a far more effective way of actually teaching and learning and with all these people if we had to give them … an hour each you’d be looking at considerably more time which I feel would be less well spent, in actual fact.” (GP Supervisor 4)*

*“It’s ridiculous to waste the hours saying right you’re just a medical student so we’ll do 2 hours here and we’ll do 2 hours here (teaching GP registrars).” (Practice Manager 1)*

### Facilitators

#### Supportive environment

Overwhelmingly, participants felt VI sessions in practices provided a supportive learning environment. Students and registrars indicated they felt confident in seeking assistance and articulating their knowledge gaps. The supportive environment was enhanced by quarantining group learning sessions, which participants described as an example of the General Practices’ commitment to prioritise teaching.

*“It’s a nice friendly environment to have a discussion in, you can always ask whatever question you want, you don’t feel silly, everyone’s there to learn and so that’s really good.” (GP Registrar 2)*

*“Even if you get it wrong, this is why it’s wrong, this is the right answer, this is the right approach. It’s not judgemental but this is how it’s done and improve for next time and you get really good feedback and do things right and really constructive you can improve on something.” (Medical Student 5)*

#### Collegiality

Multi-levels of learners fostered a sense of belonging and collegiality among participants. The relationship between registrars and students was identified as cultivating ‘near peer’ learning, because of the greater proximity of the registrar to the student’s stage of learning. Students and registrars accredited the supportive environment and collegiality within the clinic as essential components of a positive learning experience.

*“Research tells us that near peer learning is actually the most effective so these guys (medical students and GP registrars) probably got no more than 3 – 4 years between them and they’ve largely been through the same process at University, same University.. So they’ve got some immediate relationship going between each other.” (GP Supervisor 4)*

#### Community

General practices actively promoted their role as a teaching practice within the community. A supportive community of patients was deemed imperative in delivering quality education to learners. Patient feedback was utilised as an informal means of evaluating the skills and progress of the learner, but was also thought to evoke a sense of pride in the patients that they were involved in educating future doctors.

*“They’re the bug under the microscope and a lot of them feel very comfortable about doing that and to have the opportunity to sort of tell their story to the junior medical student or the junior doctor I think it’s beneficial to the patient as well. They feel like the clinic is interested in me and they listen to what I say.” (Practice Manager 1)*

“*They (clinic staff) give you regular feedback from patients. They say thank you so much for taking the time to explain this to me… Sometimes its better the students have got extra time to sit with patients and explain this is why the thyroid isn’t working and this is what we are doing to remediate that.” (Medical Student 5)*

### Barriers

#### Financial remuneration

Practice managers and supervisors described the financial impact of VI and LIC programs on the general practice. Educating learners within the practice and quarantining educational time was described as a cost negative, despite the overwhelming acknowledgment that VI allows for shared learning sessions and increases the efficiency of teaching. Some participants advocated that remuneration should be increased, while others felt the personal satisfaction they gained from training students and registrars counterbalanced the financial impact.

*“I mean financially there is a tangible cost…… but once again either way that is counterbalanced by the intangible benefits.” (GP Supervisor 2)*

*“Remuneration to the Supervisors and to the Practices needs to be increased. The Practice Incentive Payment which focuses on parallel teaching only, doesn’t take into the cost of actually having a student or a registrar and so any subsidy to the practice in administration time is financially not covered.” (Practice Manager 2)*

#### Learning style

Some participants did not feel that VI sessions aligned with their preferred learning style, preferring a more didactic approach. Students and registrars felt there were occasions when the shared sessions were pitched outside their learning needs. This was also acknowledged by supervisors as a barrier.

*“I mean everything you could just read and study. I don’t find much of a benefit presenting on cases.” (GP Registrar 1)*

*“You don’t really know if you’ve always met their learning needs because in a large group…, they might fly under the radar a bit.” (GP Supervisor 1)*

#### Group dynamics

Participants identified disruptive or unengaged learners within a group, however infrequent, proved counterproductive to VI. In such situations supervisors indicated that they employed adult learning techniques and alternate learning formats to mitigate the negative impact on other learners. Students identified that learning barriers arose when more senior learners were not open and transparent within the group when there was a gap in their knowledge.

*“(If) there is a clash in personality and it’s not an open format of learning going in multi directions then I suppose it could be a problem.” (Medical Student, 5)*

*“We had one occasion when a registrar and I can’t think whether he was GPT2 at the time was quite disruptive within the group, very disengaged, thought he knew everything.” (GP Supervisor 4)*

## Discussion

Participants in this study had difficulty defining VI precisely, however they were able to recognise it occurring in the context of their rural LIC program in the form of shared learning sessions with other levels of learners in the practice, parallel consulting and informal interactions with clinical staff. There is an opportunity to provide learners with clearer definition of the purpose, structures and opportunities for VI in the rural General Practice/LIC program context, with the potential to empower learners to become more actively engaged in the process of articulating their learning needs and maximising their VI experience [3, 33].

In alignment with previous research outside an LIC context, participants expressed that all levels of learners can benefit from VI, recognising that each level of learner has unique knowledge and skills to share [12, 14, 34]. This sharing of teaching and learning roles may be particularly enhanced where LIC programs occur, as over time students have been found to develop strong trusting relationships with their supervisors, colleagues and patients [15, 18]. LIC students have been found to feel more comfortable and confident in revealing their limitations and learning goals to their supervisors than students in block rotations, who have less contact with their supervisor and therefore may feel more pressured to perform when in their presence [35]. Similarly, learning is enhanced when supervisors are comfortable revealing their own limitations and continued learning needs [15]. The participants’ comments relating to the supportive and collegial environment they experienced during group sessions support the notion that the LIC context is enhancing these aspects of VI learning.

Clearer structuring of learning objectives, format and content for VI sessions in General Practices is a critical area for improvement identified by this study. Participants requested a resource clarifying specific learning objectives for each level of learner. Resources developed for other contexts, such as Crossley’s roadmap describing the developmental levels from student to experienced doctor, could be applied in the VI context to provide a developmental roadmap for learning applicable across the continuum of medical training [36, 37].

Evaluation of VI sessions was an area for improvement identified by participants. Effective evaluation of VI in this context will require the development of a shared understanding amongst participants of its purpose(s), against which it can be evaluated [3]. Evaluation of educational objectives will require understanding and alignment with the learning requirements of the varied participants. The scope of VI activities to be included in the evaluation will require clear definition, given the varied perceptions of what constitutes VI. A realist approach to evaluation that seeks to understand how and why VI works in LIC programs will be important to allow for the extrapolation of findings into other contexts [38].

The cost/benefit conjecture surrounding VI was raised by participants in the study and has previously been cited in the literature as a concern [12, 14, 33]. Programs with longitudinal attachments of students, such as LICs, have been suggested to be less costly than short term rotations, with some studies describing them as cost neutral [16, 39]. Such discourse about the financial cost of VI is likely to continue until a more holistic analysis is undertaken, investigating potential financial gains within other areas of the practice from having multiple levels of learners, such as recruitment and retention of staff [22, 40].

VI would benefit from greater collaboration between training bodies along the medical education continuum to establish shared curricula and developmental learning objectives. This presents a significant practical challenge when training programs at different stages operate relatively independently, as they currently do in our context [3]. The proposed establishment of a National Rural Generalist Training Pathway in Australia may stimulate a renewed focus on VI in rural and regional areas as it seeks to create continuous pathways encompassing undergraduate, prevocational and vocational training [37]. Such collaboration between universities, health services and GP training bodies presents an ideal opportunity for the design and implementation of VI training strategies and resources to be embedded throughout the medical training continuum [37].

### Limitations

As this was a phenomenological qualitative study, 15 participants representing 8 general practices was an adequate number of participants. It would have been beneficial to gain further insights from GP registrars, but due to exams occurring at the time, recruitment was difficult. Our findings illustrate perceptions of VI within a single comprehensive rural LIC program, which may limit translation of findings to other contexts.

## Conclusion

VI was found to be occurring in General Practices hosting students participating in a comprehensive rural LIC program, but was not well recognised or defined as a learning methodology. Satisfying and efficient sharing of knowledge among learners at different stages was facilitated by the supportive and collegiate environment identified as being present in a rural LIC context. There is a need for coordination between training bodies involved in the General Practice training pathway to develop appropriate resources for planning and evaluating VI to ensure all learners’ educational needs are met. The broader impact of VI in terms of facilitating workforce and economic benefits for the community requires further exploration particularly given the concerns raised regarding the short term financial implications for practices.

## Data Availability

The data that supports the findings of this study is available from Deakin University but restrictions apply to the availability of this data, which was used under license for the current study, and so is not publicly available. Data is however available from the authors upon reasonable request and with permission of Deakin University.

## Abbreviations

ASGC RA: Australian Standard Geographical Classification: Remoteness Area
GP: General Practice
LIC: Longitudinal integrated clerkship
RCCS: Rural Community Clinical School
VI: Vertically Integrated Learning
